# Extending aquatic spectral information with the first radiometric IR-B field observations

**DOI:** 10.1093/pnasnexus/pgad340

**Published:** 2023-10-14

**Authors:** Henry F Houskeeper, Stanford B Hooker

**Affiliations:** Department of Applied Ocean Physics & Engineering, Woods Hole Oceanographic Institution, Woods Hole, 02543 MA, USA; Goddard Space Flight Center, National Aeronautics and Space Administration, Greenbelt, 20771 MD, USA

**Keywords:** aquatic optics, absolute radiometry, black-pixel, IR-B, shortwave infrared (SWIR)

## Abstract

Planetary radiometric observations enable remote sensing of biogeochemical parameters to describe spatiotemporal variability in aquatic ecosystems. For approximately the last half century, the science of aquatic radiometry has established a knowledge base using primarily, but not exclusively, visible wavelengths. Scientific subdisciplines supporting aquatic radiometry have evolved hardware, software, and procedures to maximize competency for exploiting visible wavelength information. This perspective culminates with the science requirement that visible spectral resolution must be continually increased to extract more information. Other sources of information, meanwhile, remain underexploited, particularly information from nonvisible wavelengths. Herein, absolute radiometry is used to evaluate spectral limits for deriving and exploiting aquatic data products, specifically the normalized water-leaving radiance, Γ(λ), and its derivative products. Radiometric observations presented herein are quality assured for individual wavebands, and spectral verification is conducted by analyzing celestial radiometric results, comparing agreement of above- and in-water observations at applicable wavelengths, and evaluating consistency with bio-optical models and optical theory. The results presented include the first absolute radiometric field observations of Γ(λ) within the IR-B spectral domain (i.e. spanning 1400–3000 nm), which indicate that IR-B signals confer greater and more variable flux than formerly ascribed. Black-pixel processing, a routine correction in satellite and in situ aquatic radiometry wherein a spectrum is offset corrected relative to a nonvisible waveband (often IR-B or a shorter legacy waveband) set to a null value, is shown to degrade aquatic spectra and derived biogeochemical parameters.

**Significance Statement**

Optical oceanography presently defines the radiance emitted from the water surface in the infrared domain spanning 1400–3000 nm (IR-B) to be null, and aquatic spectra are routinely offset-corrected relative to the longest observed wavelength. This study presents the first absolute radiometric observations in the IR-B domain and shows derived data products with greater amplitude plus more variable flux than was formerly ascribed. Consequently, a null approximation of IR-B signal is inconsistent with the optical properties of sunlit aquatic environments and low signal-to-noise sensors. Routine spectral-offset (black-pixel) corrections, including those using the IR-B domain, are further shown to artificially darken aquatic spectra and degrade visible (VIS) derived data products. IR-B information is shown to represent an underexploited opportunity in aquatic radiometry.

## Introduction

Sunlit radiometric observations of aquatic environments have established fundamental optical relationships to support Earth Observation System (EOS) remote sensing using visible (VIS) wavelengths (1–3) and, more recently, shorter and longer end members in the neighboring ultraviolet (UV) and infrared (IR) domains (4–12). Regional differences in VIS bio-optical relationships were previously attributed to *optical complexity*, and aquatic EOS approaches were restricted to *optically simple* ecosystems. The latter, defined as case-1 waters (2), are predominantly found in the open (deep) ocean but are also found in the coastal zone and freshwater lakes. Expanding the spectral end members that define the range of radiometric observations, i.e. exploiting the UV and IR domains, improves the robustness of EOS remote sensing (10). End-member analysis (EMA)—which leverages information from the shortest and longest available wavebands—captures a greater dynamic range of environmental variability (6, 13) and mitigates deleterious nonlinearities in VIS bio-optical relationships (9), thereby enabling globally consistent aquatic optical inversion algorithms (8, 10, 11). Despite recent spectral range expansions beyond the VIS domain, fundamental characteristics and potential radiometric applications of the aquatic light field remain incomplete and unexploited for much of the applicable electromagnetic spectrum.

Spectrally dependent absorption of electromagnetic radiation by liquid water results in maximum observable signal—derivable from a point observation or an image pixel—within (approximately) the VIS domain (14). Neighboring UV and IR spectral subdivisions are defined in nanometers (nm) as the UV-A (315–400 nm) and IR-A (780–1400 nm) domains (15)—although such classifications are not universal—and are characterized by lower signals that are nonetheless measurable using commercial-off-the-shelf (COTS) sensors. Shorter wavelengths, e.g. UV-B (280–315 nm), constitute substantially lower signal than UV-A due to increased liquid water absorption and decreased atmospheric transmission, with the lower end reasonably considered as inapplicable for COTS technologies. Longer wavelengths, e.g. IR-B (1400–3000 nm), constitute lower but variable signal compared to IR-A due to water absorption and atmospheric transmission but are demonstrated herein to be observable using COTS technology. Consequently, the IR domain provides a potential opportunity for spectral range expansion in aquatic radiometry while retaining a sufficiently high (i.e. *bright*) signal or flux for COTS measurements. Expanding the spectral range of aquatic data products may support *fit-for-purpose* (11) optical inversion algorithms to advance characterization of aquatic constituents often attributed to optical complexity (e.g. inorganic particles).

Radiometric observations have previously characterized IR variability for bright aquatic near-surface features, including foam, bubbles, and algae (12, 16–18). For aquatic targets not brightened by heterogeneic near-surface features, aquatic field observations are based on deriving the flux emanating from a sunlit water body with an unobstructed surface expression, and correcting for reflected light, i.e. glint (19), to derive the water-leaving radiance, $L_{W}$. Spectral observations of $L_{W}(\lambda )$ are normalized by the global solar irradiance, $E_{s}(\lambda )$, to yield the remote sensing reflectance, $R_{\mathrm{rs}}(\lambda )$. The $R_{\mathrm{rs}}(\lambda )$ term is then adjusted with the time-dependent mean extraterrestrial solar irradiance (derived from look-up tables), $F_{0}(\lambda ,t)$, to yield the normalized water-leaving radiance, denoted Γ(λ) hereafter (12), as follows:


(1) $$\Gamma (\lambda )=F_{0}(\lambda ,t)R_{\mathrm{rs}}(\lambda ).$$

Characteristics of Γ(λ) at IR-B wavelengths are presently unknown (20) and are considered negligible (i.e. *black*) due to signal limitations (21) despite a paucity of in situ observations. Satellite, airborne, and in situ spectra using pixel or discrete observations are frequently offset-corrected by subtracting a selected long wavelength (IR-A or IR-B) signal, i.e. black-pixel processing, thereby setting the spectral end member to null and removing it to yield a *relative* spectrum.

Historical Γ(λ) field observations were primarily obtained using relative spectra, wherein observations were normalized by waveband ratios or differences to ensure physical (positive flux) results (22). For example, airborne and in situ offset-corrected IR-A anomalies at 1020 and 1071 nm have been leveraged to improve linearity of total suspended matter (TSM) algorithms for turbid estuaries (5, 7). Further spectral range expansions add *darker*, or more signal-limited, regions of the electromagnetic domain, wherein some COTS instruments frequently produce negative (nonphysical) Γ(λ) estimates at spectral end members (23). One approach to overcome the reduced flux challenges while maintaining data product efficacy is to adopt an *absolute* radiometry perspective, wherein the physical, electrical, and optomechanical parameters of the instrumentation are thoroughly characterized, while adhering to traceable standards and appropriate corrections, such that observations and derived spectral products conform to an absolute radiation scale (22, 24).

In this study, absolute radiometric observations obtained using analog and digital COTS instrumentation with consistent architectures, components, and processing principles are presented for an expansive spectral range of 313–1640 nm (UV-B to IR-B). This range is more than 1300 nm wide (compared to the legacy VIS domain width of 300 nm) and adds initial aquatic observations in the IR-B domain (25–27). Instrument components are similar for all wavebands except that silicon photodiode (SiP) detector subcomponents transition to indium gallium arsenide (InGaAs) photodiodes for longer and wider wavebands (greater than 10 nm), where appropriate. To substantiate absolute responsivity in the field across more than one of the three gain stages for all wavebands, observations of celestial targets with known spectral properties are included. Confirming that all wavebands are individually quality assured supports verification (28, 29) of IR-A and IR-B observations for targets with substantially unknown spectral properties, including aquatic environments, for which there is presently inadequate validation data that matches the performance of the instruments used herein. The analyzed datasets were obtained opportunistically and constitute an environmental stability gradient spanning illumination conditions (solar to lunar, and clear sky to overcast), variable trophic levels (oligotrophic to eutrophic chlorophyll *a*, hereafter Chl *a*, concentrations), and extreme aquatic ecosystems (elevated anthropogenic sources from agricultural chemicals to wildfire ash inputs). Relative radiometric analyses are also included, when appropriate, to provide additional detail for spectral shape comparisons.

## Results and discussion

### Celestial radiometry establishes UV-A to IR-B efficacy

**Fig. 1.**
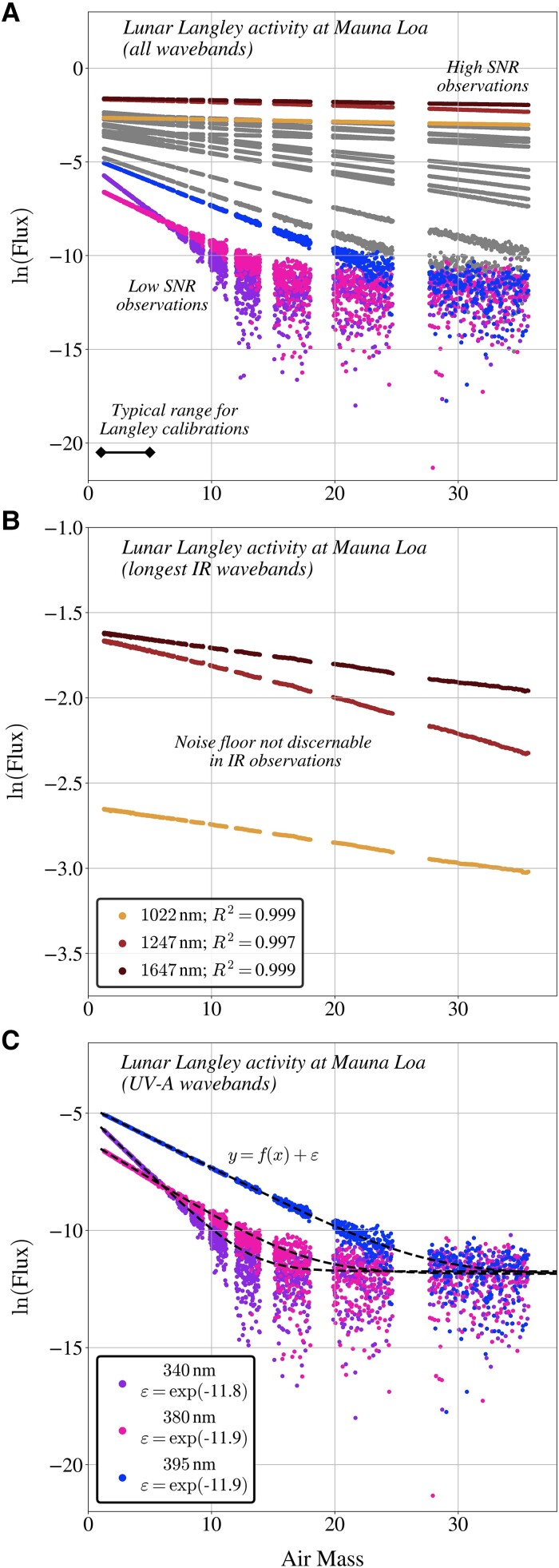
Moon-pointing radiometric observations obtained during a lunar Langley calibration activity at Mauna Loa, Hawaii. Observations from VIS to IR-A (up to 1000 nm) domains are shown in gray, observations from UV-A wavebands 340, 380, and 395 nm are shown in purple, pink, and blue, respectively, and observations from IR-A and IR-B wavebands 1022, 1247, and 1647 nm are shown in orange, red, and maroon, respectively. All waveband observations are presented in panel A, whereas the subset of observations from longer IR and UV-A wavebands are isolated in panels B and C for clarity. Linear additive noise contributions (ε) are modeled for UV-A observations in panel C and are overlaid as dashed black lines.

Lunar Langley calibrations were obtained in high-altitude pristine atmospheric conditions (Fig. 1A) for wavebands spanning UV-A to IR-B and extended far beyond the typical 1–5 air mass range to capture maximum dynamic range in instrument signal. All waveband observations adhered to log-linear relationships as a function of air mass. The lowest flux observations (UV-A for high air mass geometries) enabled estimation of an instrument noise floor (i.e. one signal-to-noise, hereafter SNR, equivalent) approximately two orders of magnitude below the aquatic observations presented herein, including the darkest oligotrophic waters.

Solar Langley calibrations were also obtained for high-altitude pristine atmospheric conditions (Fig. S1) for wavebands spanning UV-A to IR-B and verify similar digital instrument performance for all wavebands based on consistent linear responsivity, no discernible gain-stage transitions, no degradation in linearity due to stray light effects, and normally distributed Langley extrapolation residuals. Laboratory calibration enabled independent estimation of the top-of-atmosphere solar flux using Langley extrapolations, which resulted in median agreement within 2.5% for all wavebands and less than 1.5% for IR wavebands, i.e. to within the absolute calibration uncertainty (26).

The Langley activities demonstrate approximately ten decades in linear responsivity of the deployed technologies, consistent with prior analyses (11), and indicate similar performance metrics for all (independently derived) wavebands. Compliance of each independent waveband adheres to the absolute radiometry perspective, wherein waveband observations are individually quality assured and not interdependent. Celestial radiometry results are directly applicable to aquatic radiometry results presented below because instrument technologies are identical except for minor differences in the foreoptics (e.g. field-of-view, hereafter FOV) and waveband configurations.

### Aquatic IR-B flux is not signal-limited

**Fig. 2.**
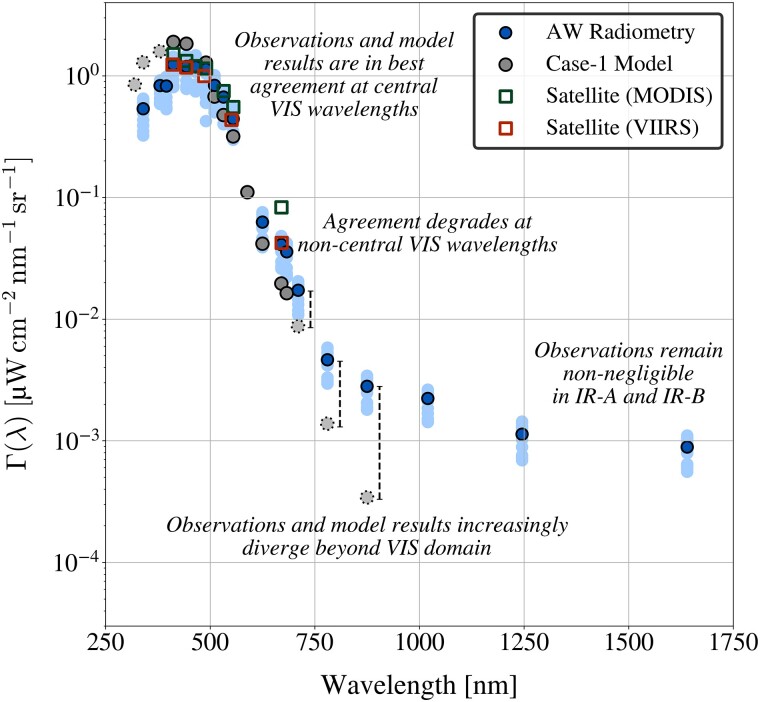
Southern Ocean minimum-to-maximum Γ(λ) observations derived using analog AW radiometry are shown as light blue shaded circles, with median waveband values overlaid as dark blue shaded circles. Neighboring mean satellite observations are shown as green and red open squares for the MODerate resolution Imaging Spectroradiometer (MODIS) and the Visible Infrared Imaging Radiometer Suite (VIIRS), respectively. Satellite average values were used for Southern Ocean characterization because the observations were collected during fully overcast conditions. Modeled Γ(λ) derived using optically simple (case-1) parameterizations is shown as gray shaded circles, with solid or dotted borders indicating quality assured (VIS) or out-of-bounds (UV-A and IR-A) model parameterizations.

Above-water (AW) in situ radiometric observations of Γ(λ) were obtained for oligotrophic Southern Ocean waters using an analog radiometric suite that was a prototype for the previously described digital celestial radiometers and relied on similar hardware, including optical subcomponents. The Southern Ocean Γ(λ) spectra span the UV-A to IR-B domains (Fig. 2), and satellite spectra from neighboring waters are in close agreement with applicable (VIS) observations (Pearson correlation coefficient, *r*, for the median waveband values was near unity). Consistent with the celestial radiometric instrumentation, all wavebands were individually quality assured and used identical acquisition and processing software plus similar hardware (with the exception of the SiP and InGaAs transition). Aquatic IR-A and IR-B observations were between one and two orders of magnitude greater than the noise floor derived using lunar radiometry.

**Fig. 3.**
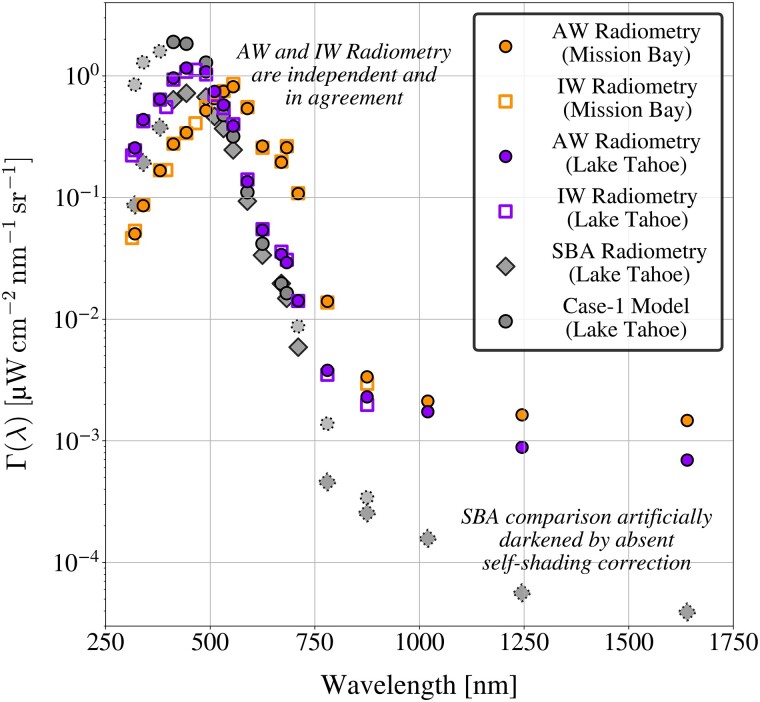
AW and IW radiometric derivations of Γ(λ) are shown as shaded circles and open squares, respectively, for observations obtained in situ at Mission Bay, California (orange), and Lake Tahoe, California and Nevada (purple). AW radiometric observations were obtained in careful adherence to community protocols (31). Radiometric observations obtained at Lake Tahoe using the skylight-blocked approach (32), termed SBA, are shown as gray diamonds for spectral shape comparison, but are artificially darkened due to the absence of a self-shading correction applicable beyond 405–720 nm (dotted borders). Modeled Γ(λ) derived using optically simple (case-1) parameterizations is shown as gray shaded circles, with solid or dotted borders indicating quality assured (VIS) or out-of-bounds (UV-A and IR-A) model parameterizations.

The analog radiometry suite deployed in the Southern Ocean was succeeded by a compact COTS instrument design, wherein analog circuitry was replaced with digital microradiometers (30), and subsequent Γ(λ) observations were obtained using contemporaneous AW and in-water (IW) radiometry. Figure 3 shows agreement of Γ(λ) observations derived using AW and IW radiometry at applicable wavelengths, with observations obtained at an oligotrophic alpine lake (Lake Tahoe) and a eutrophic tidal bay (Mission Bay). An alternate AW data acquisition method (32), i.e. the so-called skylight-blocked approach (SBA), also independently verifies spectral shape for the SBA and AW Lake Tahoe observations, although the SBA radiometric observations are artificially darkened due to the absence of an applicable SBA self-shading correction beyond 405–720 nm (32).

The Southern Ocean and Lake Tahoe surface radiometry activities sampled aquatic environments that were similarly oligotrophic (Chl *a* < 0.08 mg m${}^{-3}$), conservative (8), and optically simple (2). Bio-optical parameterizations determined from case-1 relationships would predict similar optical properties of the two water bodies (33). In keeping with established case-1 theory, the surface radiometry activities produced consistent Γ(λ) values at IR wavelengths despite using different instruments (i.e. analog versus digital), providing an additional verification of the IR data products.

Observations of Mission Bay were characterized by Chl *a* values that were approximately two orders of magnitude greater than at Lake Tahoe and the Southern Ocean. Consistent with higher anticipated organic particle content attributed to higher Chl *a*, the IR-A and IR-B Γ(λ) observations at Mission Bay were approximately two times greater—and UV-A observations approximately half as great—as the magnitude of those observed at Lake Tahoe. Lake Tahoe observations adhere to optically simple (case-1) conditions according to Γ(λ) characteristics that were consistent with a legacy case-1 model at applicable wavelengths (10, 33).

The Southern Ocean, Lake Tahoe, and Mission Bay results challenge the assumption that Γ(λ) signals are negligible within the spectral range assessed herein (i.e. 313–1640 nm), even for optically simple, case-1 water bodies (21, 34). Based on SNR values tabulated for forthcoming next-generation oceanographic satellite sensors (35), the IR-B AW radiometric observations in the Southern Ocean, Lake Tahoe, and Mission Bay are greater than the anticipated noise floor.

Within a black-pixel perspective, aquatic spectra (in situ, airborne, or satellite) are offset-corrected to set the assumed negligible IR-B signals to zero. If the Lake Tahoe and Mission Bay Γ(λ) observations were offset-corrected (i.e. the magnitude of the 1640 nm data product was subtracted from the rest of the spectra), darkening in the 670 nm data product would correspond to 41% and 15%, respectively, of a 5% proposed next-generation uncertainty target (35). IR-B offset corrections deleteriously darken derived AW Γ(λ) spectra and degrade agreement between IW and AW observations. Artificial darkening propagates to higher-level data products; for example, an IR-B black-pixel correction reduced a biogeochemical parameter estimated using above-water observations and a globally robust EMA algorithm (10) by 4.3% and 10.3% at Mission Bay and Lake Tahoe, respectively.

### Spectrally expansive observations confer maximal information

**Fig. 4.**
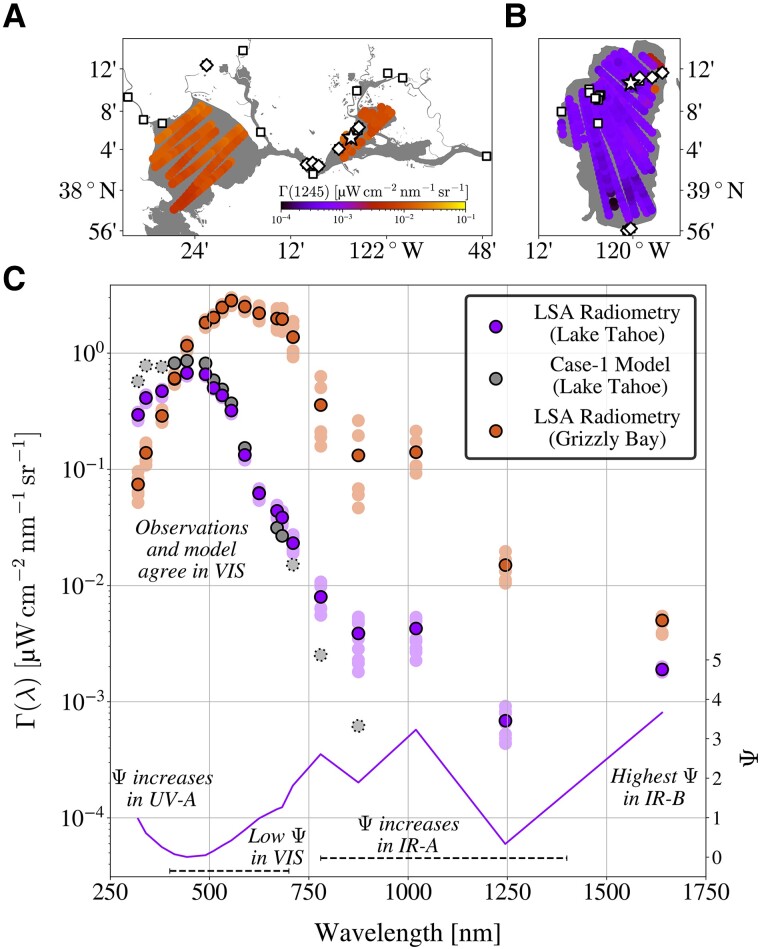
Airborne remote sensing observations obtained at lowest safe altitude (LSA). Enlarged surface spot observations of Γ(1245) are shown using a unified $\log_{10}$ color scale for San Pablo and Grizzly Bays, California (panel A), plus Lake Tahoe (panel B). Locations of in situ water sampling are shown as white diamonds and squares for contemporaneous and noncontemporaneous field observations, respectively. Panel C shows Γ(λ) spectra from LSA radiometry obtained at contemporaneous field sites indicated by white stars in panels A and B. Spectra obtained at Lake Tahoe and Grizzly Bay are shown as light purple and light orange filled circles, respectively, with median corresponding spectra overlaid in dark purple and dark orange. Modeled Γ(λ) derived using optically simple (case-1) parameterizations is shown as gray shaded circles, with solid or dotted borders indicating quality assured (VIS) or out-of-bounds (UV-A and IR-A) model parameterizations. A peak-normalized difference spectrum (12), Ψ(λ), is shown corresponding on the right *y*-axis.

Wildfire smoke and ash modify the bio-optical characteristics of aquatic ecosystems (36), including through the mitigation of photoinhibition, injection of nutrients into near-surface waters, and increased productivity. Airborne remote sensing surveys of Lake Tahoe plus San Pablo and Grizzly Bays, California, during active wildfires indicated that IR-B variability between sites was reduced during sampling in the presence of nearby fires with concomitant windblown trails of smoke and ash, whereas IR-A observations maintained order-of-magnitude differences between sites. Airborne surveys of Γ(1245) values are shown in Fig. 4 along with representative spectra showing decreased inter-site variability in Γ(1640).

The active wildfire season observations indicate Γ(λ) blue and red edges, as well as local minima and maxima in the IR domain, in keeping with optical complexity modes (12) and previous optical complexity scenarios involving whitecaps and algal surface expressions (16–18). Lake Tahoe field observations of Chl *a* and airborne observations of Γ(λ) were mostly elevated during the active wildfire season relative to prior baseline sampling, with the greatest relative increase in Γ(λ) recorded for the IR-B waveband. This result is consistent with shallower optical depth for IR-B data products, which increases sensitivity to near-surface components (e.g. settling wildfire ash), combined with previous evidence that brightening of the IR waveband most spectrally separated from the VIS domain corresponds to increasing optical complexity, wherein complexity is defined as a continuum rather than a binary partitioning of water bodies (12).

## Conclusions

The term *negligible*, as applied here to the legacy treatment of IR-B aquatic signals, indicates a signal-limited observation, i.e. the flux is below the in situ or satellite threshold for deriving an accurate Γ(λ) data product. It also indicates a sufficiently small flux, wherein an offset correction at a spectral end member—for example, within an atmospheric correction scheme (21)—would not deleteriously alter the optical characterization of the environment. The results presented herein document non-negligible aquatic Γ(λ) signals spanning 313–1640 nm for optically complex (inland shallow) waters as well as optically simple (oligotrophic deep) waters and challenge present knowledge of aquatic light environments, in which IR-B signals—as well as IR-A in deep oligotrophic environments—were formerly assigned as negligible. Values reported herein—which accounted for stray light and out-of-band effects, dark current variability, and stochastic glint spikes—are non-negligible based on the detector technologies and processing employed, both of which support adherence to an absolute radiometric scale.

The signals observed are greater than predicted using pure-water models—anticipated because Γ(1640) values correspond to a very near-surface layer, including the surface microlayer (SML), in which organic and inorganic components are highly enriched (37–40). Adding information from spectral domains (i.e. the IR-B) previously considered to confer null signal constitutes an opportunity to advance aquatic remote sensing. Models to estimate Γ(λ)—including radiative transfer (41) or quasi-single scattering approximations (42) based on water absorption (43)—were not intended for application to the IR-B domain. Both the AW and SBA results presented herein for the IR-B waveband are brighter than would be predicted by modeling, indicating that the model results are possibly nonphysical (SBA observations prevent glint—including skylight—contamination and constitute a lower boundary, albeit with artificial darkening from self-shading). Future work should continue to investigate the differences between the modeled values and these initial IR-B observations.

Potentially viable next-generation strategies for advancing aquatic remote sensing include, but are not limited to, the following: increasing the spectral domain (e.g. EMA); increasing the spectral resolution of observations (i.e. hyperspectral); and adding new parameters (e.g. polarimetry). Hyperspectral sensing increases the number of data products available for extracting information, albeit at the expense of radiometric accuracy (29) and with high correlation between spectrally adjacent wavebands (12, 44). Extraction of polarization information may improve atmospheric correction (45) and enable new applications (46). Hyperspectral polarimetry is included on a forthcoming oceanographic satellite (47). Implementation of the latter two strategies does not include IR-B field observations, so only increasing the spectral domain is considered hereafter.

### Leveraging optical observations

Additions of new wavelength domains have been shown to improve aquatic algorithms by increasing robustness in the presence of optical complexity and by mitigating regional differences in VIS bio-optical relationships (8, 10). The observations presented herein demonstrate that a more expansive spectral range than formerly leveraged, i.e. 313–1640 nm, is applicable to aquatic radiometry and observable using COTS technologies. The use of COTS instruments ensures the community of practice can leverage expanded spectral range to further aquatic research. The noise floor derived by considering lunar Langley activities was less than the aquatic Γ(λ) signals derived from UV-B to IR-B domains, in agreement with a recent geostatistical SNR analysis (23). Performance of individual wavebands was similar and objective, which supports verification of Γ(λ) spectra using contemporaneous IW radiometry, albeit with a lesser spectral range.

### Aquatic IR-B characteristics

Forthcoming ocean-observing satellites are anticipated to continue to include black-pixel processing schemes for atmospheric correction (45). Black-pixel processing schemes rely on sensor SNR characteristics, and black-pixel wavebands must be sequentially moved to longer wavelengths as technology advances (48), as formerly acceptable (but poor) uncertainty requirements are revised to meet new and evolving science objectives (48), and as the diversity of targeted environments expands, e.g. to support applications to more turbid or high-biomass waters (49). Consequently, such methods—which at present are routinely used for in situ, airborne, and satellite observations—are inconsistent with the optical properties of aquatic ecosystems as the state of the art inexorably advances.

Insufficient historical field observations revealing IR-B variability in natural waters (20) are attributable to in situ instrumentation challenges, in part, because hyperspectral spectrometers—required to support next-generation hyperspectral aquatic science objectives—optimize wavelength resolution at the expense of sampling rate and dynamic range, which degrades sensitivity and spectral range (29). The IR-B (plus IR-A, UV-B, and UV-A) signals derived herein are not negligible from a next-generation remote sensing perspective, e.g. signal amplitudes exceed the anticipated noise floor (i.e. oligotrophic clear-water observations are approximately four to five times greater than proposed noise requirements) for forthcoming missions (35). However, demonstration of non-negligible Γ(λ) values in the IR-A and IR-B domains shown herein challenges the utility of a black-pixel approach regardless of whether forthcoming sensors can accurately resolve IR-B signals: small biases based on *a priori* but nonphysical assumptions degrade data products in other, e.g. VIS, spectral domains.

### Exploiting aquatic variability

Although the radiometric observations presented herein challenge common assumptions about aquatic light fields at IR wavelengths, the results are consistent with fundamental bio-optical principles, as follows: spectral agreement was verified between optically simple waters with similar Chl *a* content (consistent with case-1 theory); improved algorithmic relationships have previously been demonstrated for UV-B, UV-A, and IR-A data products (8–11); and increased IR signals were observed in environments expected to contain high particle content. The latter is consistent with prior reports of elevated IR-A signals in turbid waters (5, 7, 10, 50, 51). Variability in IR-B optical properties represents an underexploited analytical opportunity in aquatic in situ and remote sensing radiometry. For example, the maximum Fig. 4 spectral mode amplitude is in the IR-B end member. Black-pixel offset corrections evaluated herein removed the information corresponding to the highest amplitude spectral mode (12), produced nonphysical (negative) data products, and degraded Γ(λ) spectra based on AW and IW comparisons. Artificial darkening due to an IR-B offset correction was most pronounced in the red and IR-A wavelength domains, wherein IR-A wavebands have previously been shown to support globally consistent aquatic optical inversion algorithms (10, 11). Developing globally consistent applications in aquatic radiometry requires preservation of expansive spectral range information (8).

## Materials and methods

The observations used herein were collected during field campaigns spanning approximately 7 years. The instrument technologies leveraged continuing advances in hardware, software, and field capabilities for AW and IW radiometry, with the former including celestial radiometry. In each field deployment, the instrument suites were in a new or prototypical configuration, and the data obtained provided partial confirmation of software and hardware performance metrics. Because performance compliance was obtained in all cases, the datasets are presented herein, as follows: (a) solar and lunar observations at Mauna Loa Observatory (Hawaii) and in near proximity to the Mount Laguna Observatory (California); (b) in situ aquatic observations in the Southern Ocean, plus the inland waters of Mission Bay (California) and Lake Tahoe (California and Nevada); and (c) airborne aquatic surveys during active wildfires of Grizzly Bay (California; part of the westernmost extent of the California Delta), San Pablo Bay (California; immediately north of San Francisco Bay and connected to Grizzly Bay by the Carquinez Strait), and Lake Tahoe.

### Optical instruments

Quantification of spectrally expansive Γ(λ) data products is the primary objective herein, and therefore AW (rather than IW) radiometers are the primary focus because low but measurable UV-A, IR-A, and IR-B signals are maximum above the water surface. Solar- and lunar-pointing radiometers plus submersible aquatic radiometers are included to establish technological performance metrics and provide verification including AW and IW spectral comparisons for compliant wavebands, e.g. 320–875 nm (11, 12, 28, 29). Verification is important for two reasons: (a) there is presently inadequate IR-B validation data matching the performance of the instruments used herein; and (b) the celestial radiometry and AW aquatic methods are purely objective and identical for all wavebands, so proven efficacy within the UV-A to IR-A domains is applicable to the IR-B domain.

####  

##### Celestial radiometric instrumentation

Celestial radiometric instrumentation consisted of the Optical Sensors for Planetary Radiant Energy (OSPREy) prototype and the newest compact generation of the instrument suites (C-OSPREy), which were developed to support expansive spectral- and dynamic-range radiance or irradiance observations (plus three-axis polarimetry for the latter) of solar, lunar, sky, and aquatic targets (27, 52). The OSPREy and C-OSPREy instrument suites include a removable shroud to mitigate stray light effects, an improved irradiance diffuser compliant across UV-B to IR-B domains, and a narrow FOV angle ($2.5^{\circ }$) to support celestial radiometry. Although both instrument suites share significant common architectural and technological components, the next-generation C-OSPREy suite improves the capabilities of the OSPREy prototype and uses a compact form factor to reduce instrument size by 33% (27). Although the OSPREy and C-OSPREy technologies are presented herein only for celestial radiometric applications, both systems are also configurable to support aquatic radiometry (27). Waveband configurations for both instruments include UV-A to IR-B wavebands and are summarized in Table S1.

##### Above-water instrumentation

AW instrument suites presented herein include the Compact-Airborne Environmental Radiometers for Oceanography (C-AERO) system (11), plus the Biospherical Surface Ocean Reflectance System (BioSORS) prototype (30). Both technologies were developed to expand the spectral (and therefore dynamic) range of optical measurements and are based primarily, but not exclusively, on SiP technology. Both instruments have 10 decades of linear dynamic range, but BioSORS instruments are built with a significant number of analog components (e.g. cabling), whereas C-AERO instruments are built with digital microradiometer arrays wherein analog circuitry is miniaturized (and shielded). BioSORS is used predominantly as a testbed, e.g. to test the use of InGaAs photodiodes for wavelengths exceeding 900 nm. BioSORS supports 19 wavebands with an FOV of $6.5^{\circ }$. C-AERO measures the total surface radiance, $L_{T}(\lambda )$, the indirect sky radiance, $L_{i}(\lambda )$, and the global solar irradiance, $E_{s}(\lambda )$ simultaneously at 19 microradiometer wavebands with an FOV for the radiance radiometers of $2.5^{\circ }$. C-AERO significantly advances the capabilities of BioSORS, in pertinent part, by enabling increased sampling rates to support superior glint discretization and pointing accuracy, mitigating stray light effects through the addition of removable shrouds, and improving dark current characterization with spectrally and responsivity dependent predictive dark-current (PDC) methods (10, 11, 53). Regarding stray light effects, full spectral responsivity characterizations were performed for the fully integrated assembly using a double monochromator with prism predisperser, equipped with three gratings for covering a wavelength range from 200 to 2000 nm. Reference detectors traceable to the National Institute for Standards and Technology (NIST) allow accurate spectral characterizations between 250 and 1800 nm. Examination of the data from the 1640 nm wavebands from the instrument suite in question shows no evidence of quantifiable spectral leakage. Glint discretization was supported by high sampling rates (C-AERO supports sampling at up to 30 Hz), which enabled discretization (and rejection) of glint spikes. Glint filtering ensures derived data products are free from glint contamination, which is difficult to remove from legacy (e.g. 0.5 Hz) observations that temporally average stochastic and non-Gaussian glint contributions (10, 53, 54). Waveband configurations for both instruments include UV-A to IR-B wavebands (Table S1).

##### In-water instrumentation

IW instrumentation presented herein for verification purposes is the Compact-Hybridspectral Radiometer (C-HyR), a prototype handheld profiler consisting of upward-pointing irradiance and downward-pointing radiance radiometers, each with 19 microradiometer channels spanning the UV-B to IR-A domains (Table S1), as well as a downward-pointing hyperspectral spectrograph spanning 350–900 nm (25). Tilt compliance is maintained by a kite-shaped backplane with hydrobaric buoyancy and kinematic ballast, as well as by digital thrusters, i.e. the Compact-Propulsion Option for Profiling Systems (C-PrOPS), which ensure planar orientation of the profiler at the initiation of each vertical profile. C-PrOPS also improves the efficacy of IW observations by reducing cycle times between profiles, and by enabling navigation of the profiler away from a ship or dock to mitigate adjacency effects (8, 25).

### Data products

Multispectral data products are presented corresponding to observations obtained using the celestial plus AW and IW radiometric instruments. Methods for deriving data products are presented separately for celestial and aquatic radiometers. OSPREy, C-OSPREy, and C-HyR also support hyperspectral data (300–900 nm), which although not presented, provided confirmation during data processing of the efficacy of the UV-A to IR-A multispectral data products where possible.

####  

##### Celestial radiometric data products

The primary celestial radiometric data products presented herein, which are applicable to deriving atmospheric data products, are the natural log values of instrument flux, or signal, observed by an irradiance radiometer tracking the Sun or Moon. The celestial observations were obtained to support solar and lunar Langley calibration analyses while simultaneously testing instrument and auxiliary (e.g. automated solar- or lunar-tracking technology) system performance. The results of the latter are published (26, 27, 52) and not discussed further herein.

Celestial observations establish instrumentation performance metrics by deriving ordinary least-squares (OLS) fits and coefficient of determination ($R^{2}$) statistics as a function of air mass and multispectral instrument flux values transformed onto the natural-log scale. OLS residuals were tested for normality using the Shapiro-Wilk test (*W*), implemented using the SciPy Python programming library. Lunar Langley observations were conducted across an expansive range in air masses, and waveband observations indicating the instrument noise floor parameterized a model relating flux and air mass, of the form:


(2) y=f(x)+ε,

in which *y* corresponds to the observed instrument flux, the f(x) function represents the natural log-linear relationship between air mass and flux for high-signal observations, and the uncertainty term ε approximates the central tendency of the noise-floor contribution. Noise floor characteristics relevant to AW radiometry were approximated using first-order-first-moment (FOFM) estimation (55).

##### Aquatic ecosystem data products.

Formulations for producing $L_{W}(\lambda )$ are described in the literature for both AW (19) and IW (31, 56, 57) approaches, and the methodology used herein is consistent with recent publications leveraging coincident AW and IW data products to describe global aquatic optical relationships (10–12). Methodology used herein for deriving AW and IW data products requires stable illumination, with the solar disc completely occluded or completely unobstructed.

AW radiometric observations of $L_{T}(\lambda )$ and $L_{i}(\lambda )$ enable derivation of $L_{W}(\lambda )$ following Mobley (19), with geometrical terms removed for brevity, as follows:


(3) $${\hat{L}}_{W}(\lambda )=L_{T}(\lambda )-\rho (\lambda ,W_{\;s})L_{i}(\lambda ),$$

where the circumflex (L^) accent denotes an AW aquatic data product and the surface reflectance $\rho (\lambda ,W_{\;s})$ is a function of wind speed, $W_{\;s}$, measured at the water surface to parameterize *ρ* as a function of surface roughness.

An alternate AW method, termed the skylight-blocked approach (SBA), was performed by attaching a cone to the sensing end of a nadir-pointing radiometer, and submerging the edge of the cone. The rationale of SBA is that by blocking surface-reflected light, $L_{W}(\lambda )$ can be derived directly without requiring sky reflectance or water surface roughness estimates (32). The SBA approach utilizes a self-shading correction that is presently limited approximately to the VIS domain (405–720 nm), but SBA is included herein to provide spectral shape comparisons based on a separate, independent AW methodology, and to provide a lower (i.e. glint free) boundary estimate.

IW radiometric observations of upwelling radiance, $L_{u}(z,\lambda )$, recorded using a nadir-pointing radiometer profiling within a near-surface depth interval of approximately 1 m with homogeneous properties, enable the IW derivation of $L_{W}(\lambda )$. Briefly, $L_{u}(z,\lambda )$ is extrapolated to null depth, $L_{u}(0^{-},\lambda )$, and propagated through the water surface following Mobley (19), with geometrical terms removed for brevity, as follows:


(4) $${\tilde{L}}_{W}(\lambda )=0.54L_{u}(0^{-},\lambda ),$$

where the tilde (L~) accent denotes an IW aquatic data product.

### Spectral comparisons

Spectral shape comparisons between two observations of similar water masses are presented using *r* to quantify agreement between two related Γ(λ) spectra for common wavelengths (12), with *λ* terms dropped for brevity, as follows:


(5) $$r=\frac{\mathrm{cov}(\Gamma_{r},\Gamma_{c})}{\sigma (\Gamma_{r})\sigma (\Gamma_{c})},$$

where cov is the covariance, *σ* is the standard deviation, the $\Gamma_{r}$ and $\Gamma_{c}$ terms denote reference and comparison spectra, respectively, and the computation is made for all common wavelengths.

Comparisons between two related, but not identical, water masses (e.g. observations obtained before and after initiation of wildfire conditions) are presented using the relative peak-normalized difference spectrum, Ψ(λ), following Hooker et al. (12). Values of Ψ(λ) are derived by computing the relative difference between peak-normalized Γ(λ) spectra, denoted $\Gamma^{p}(\lambda )$, as follows:


(6) $$\Psi (\lambda_{i})=\frac{\Gamma_{c}^{p}(\lambda_{i})-\Gamma_{r}^{p}(\lambda_{i})}{\Gamma_{r}^{p}(\lambda_{i})},$$

where $\lambda_{i}$ denotes an individual waveband and the $\Gamma_{r}^{p}(\lambda_{i})$ and $\Gamma_{c}^{p}(\lambda_{i})$ terms denote reference and comparison peak-normalized observations, respectively.

### Case-1 model

Theoretical aquatic Γ(λ) spectra were derived using a case-1 modeling approach, in which optically active constituents are approximated as a function of Chl *a* content, as presented in Houskeeper et al. (10) and Hooker et al. (12). In short, the case-1 derivation is derived using the Processing of Radiometric Observations of Seawater using Information Technologies (PROSIT) software (26) and is based on Gordon et al. (33) with relevant terms and coefficients consistent with published literature and defined in Houskeeper et al. (10). The PROSIT case-1 dataset was previously found to agree with a HydroLight model (e.g. relative percent difference, RPD, of 1.1%) within the Chl *a* range spanning 0.02–20 mg m${}^{-3}$ (10). The addition of the theoretical Γ(λ,Chla) data products enable comparisons of spectral adherence to legacy aquatic optical properties and optically simple (case-1) relationships. Not all waters are anticipated to conform to the case-1 relationships (i.e. so-called case-2 waters), but the case-1 parameterizations provide a conceptual framework for considering spectral similarities of aquatic observations presented herein. The case-1 model parameterizations used herein are most applicable to VIS wavelengths based on legacy parameterizations, but a more expansive spectral range spanning UV-A to IR-A wavelengths—consistent with the presentation in Houskeeper et al. (10) and Hooker et al. (12)—is included herein to provide an opportunity for initial comparison for neighboring VIS wavebands.

### Biogeochemical algorithms

Estimation of the absorption coefficient for colored dissolved organic matter (CDOM) at 440 nm, $a_{\mathrm{CDOM}}(440)$, was performed using the remote sensing EMA algorithm evaluated in Houskeeper et al. (10), as follows:


(7) $$a_{\mathrm{CDOM}}(440)=A[\Lambda_{\lambda_{2}}^{\lambda_{1}}{]}^{B},$$

where $\Lambda_{\lambda_{2}}^{\lambda_{1}}$ corresponds to the ratio of $\Gamma (\lambda_{1})$ over $\Gamma (\lambda_{2})$, and coefficients *A* and *B* were obtained using recent updates (11).

### Field datasets

Radiometric datasets were obtained using the same or similar technologies, with adherence to community protocols for derivation of aquatic data products (19, 31, 57). Collected field data sets include celestial radiometry, AW and IW in situ aquatic radiometry, and lowest safe altitude (LSA) airborne remote sensing of aquatic environments coincident with nearby active wildfires.

####  

##### Radiometric observations of stable celestial targets (Sun and Moon)

Solar- and lunar-pointing instrument flux observations were derived using OSPREy at Mauna Loa (nominal elevation 4,170 m) in August–September 2012 to fulfill solar and lunar Langley calibration activities. Solar Langley observations (Fig. S1) were collected between sunrise and sunset on 31 August for sensor geometries spanning an air mass range of approximately 1.0–1.7. Due to temporal variability in atmospheric conditions, only the sunrise portion of the solar Langley observations are presented, which encompass 312 observations with an air mass range of approximately 1.0–1.5. Lunar Langley observations (Fig. 1) were obtained between moonrise and midnight on 1 September for sensor geometries corresponding to a significantly more expansive air mass range of approximately 1.3–5.7, although an expanded air mass range exceeding 30 air masses was observed, resulting in 8,958 observations. Dark characterizations were performed periodically during Langley data acquisition to ensure instrument stability.

Solar-pointing instrument flux observations (Fig. S1) were also derived using C-OSPREy at Mount Laguna (nominal elevation 1,921 m) on 4 October 2017 to fulfill solar Langley calibration activities. The C-OSPREy solar Langley observations were obtained between sunrise and noon at sensor geometries corresponding to an air mass range of approximately 1.3–2.9, although the two observations nearest noon were removed due to evolution of the atmosphere. Unlike the Mauna Loa deployments, in which OSPREy was continuously tracking the solar or lunar discs, C-OSPREy was undergoing testing of solar-tracking software during the Mount Laguna deployment, and C-OSPREy was repeatedly and intentionally powered down to evaluate the fidelity and reliability to independently reacquire and track the solar disc. As a result, C-OSPREy observations are significantly less numerous than OSPREy observations, with 16 quality assured data points.

##### Above- plus in-water observations of clear stable to turbid variable aquatic ecosystems

Oceanic AW observations of Γ(λ) were obtained by manually pointing BioSORS (at the required solar geometry) aboard the research vessel R/V *Akademik Tryoshnikov* in Southern Ocean waters starting in early January 2017 for approximately two months. The BioSORS dataset was filtered to only retain observations under fully overcast and oligotrophic (Chl *a* < 0.08 mg m${}^{-3}$) conditions to mitigate uncertainties from solar geometry and spatial heterogeneity. Satellite matchups from the MODerate resolution Imaging Spectroradiometer (MODIS) and the Visible Infrared Imaging Radiometer Suite (VIIRS) were obtained from neighboring waters using default NASA Level-3 Ocean Color products obtained from the OceanColor Web (https://oceancolor.gsfc.nasa.gov/). Average satellite values are presented because direct matchups were prevented by the requirement for overcast sky states. Satellite matchups were near unity with the in situ observations, with r=0.986 and 0.991 for the MODIS and VIIRS matchups, respectively.

Inland waters AW observations of Γ(λ) were obtained by manually deploying C-AERO on 4 June 2018 from a dock in Mission Bay, and a week later from a small boat in Lake Tahoe. Lake Tahoe observations were similarly oligotrophic compared to the Southern Ocean, with Chl *a* < 0.08 mg m${}^{-3}$ at Lake Tahoe. Mission Bay observations were eutrophic, with Chl *a* spanning 3.284 to 4.554 mg m${}^{-3}$. For both deployments, C-AERO was deployed with shrouds and using a 15 Hz sampling rate with PDC corrections, and coincident IW observations were obtained using C-HyR with C-PrOPS for verification of spectral shape and amplitude for compliant UV-A to IR-A spectral domains. Water sampling of Chl *a* and $a_{\mathrm{CDOM}}(440)$ was conducted to support self-shading corrections and confirm adherence to previously a published global algorithm (8) as additional verification, respectively. Contemporaneous IW and AW radiometry produced r=0.999 at both sites. SBA observations provided independent confirmation of AW spectral shape, with r>0.999. Spectral degradation due to IR-B offset corrections was quantified using RPD, wherein the IW observations are the reference values. RPD of offset-corrected AW spectra was found to increase by a factor of 3 in both the red and IR-A domains at Mission Bay, and by factors of 3 and 30, respectively, in the red and IR-A domains at Lake Tahoe.

##### Remote sensing aquatic surveys during active wildfires (with surface validation)

Semi-automated observations were obtained by integrating C-AERO with autonomous data acquisition software onto a Twin Otter (TO) aircraft that surveyed San Pablo Bay, Grizzly Bay, and Lake Tahoe at LSA during fall 2017 as part of the NASA Coastal High Acquisition Rate Radiometers for Innovative Environmental Research (C-HARRIER) airborne campaigns (10, 53). LSA flight altitudes, as low as 100 ft or 30.5 m, remove the need for an atmospheric correction of the $L_{T}$ observations. The C-AERO instruments were integrated into the body of the TO aircraft at the required pointing geometries. C-HARRIER flight paths were designed to minimize flight altitude and ensure compliant sensor-solar geometries while sampling the target water bodies. Glint filtering of airborne data was achieved based on discretization of stochastic and non-Gaussian glint spikes, which has previously been applied to airborne remote sensing campaigns to minimize uncertainties in surface reflected light (10, 53, 54). C-HARRIER flights surveyed San Pablo and Grizzly Bays on 8 September 2017 and Lake Tahoe on 13 September 2017 in coordination with a field team that obtained coincident IW observations from a small boat in Grizzly Bay and Lake Tahoe, respectively, using C-OPS with C-PrOPS, plus coincident water sampling. Additional near-coincident field sampling was performed in Carquinez Straight (8 September 2017), plus Hudeman Slough (6 September 2017), which provides source waters to San Pablo Bay via Sonoma Creek. Additional contemporaneous field sampling for Lake Tahoe was performed at the Tahoe Keys private waterfront marina at the southern edge of Lake Tahoe (7 September 2017).

The September 2017 wildfire season was highly active in the western US, resulting in broad-scale reductions in air quality and daytime shortwave radiation (58). Airborne surveys occurred approximately one to two weeks following active wildfires in the nearby Sierra Nevada mountains of California and Nevada, which contributed smoke and ash to the water basins associated with the survey sites. Smoke plume trajectories are frequently variable due to temporal changes in local wind patterns, and one scenario is shown near Lake Tahoe on 1 September 2017 in Fig. S2.

## Supplementary Material

pgad340_Supplementary_Data

## Data Availability

The data underlying this article are available in Dryad at (59): Houskeeper, Henry; Hooker, Stanford (2023). Extending aquatic spectral information with the first radiometric IR-B field observations [Dataset]. *Dryad*. https://doi.org/10.5061/dryad.pc866t1tk
