# Supplementary material for: Extending aquatic spectral information with the first radiometric IR-B field observations
Source: PNAS Nexus. 2023 Oct 14;2(11):pgad340. doi: 10.1093/pnasnexus/pgad340 (PMC10627478; doi:10.1093/pnasnexus/pgad340)
Supplement: pgad340_Supplementary_Data [file pgad340_supplementary_data.pdf]

Supporting Information for

Extending aquatic spectral information with the first radiometric IR-B field observations

Henry F. Houskeeper and Stanford B. Hooker

Henry F. Houskeeper.
E-mail: henry.houskeeper@whoi.edu

This PDF file includes:

Figs. S1 to S2
Table S1
SI References

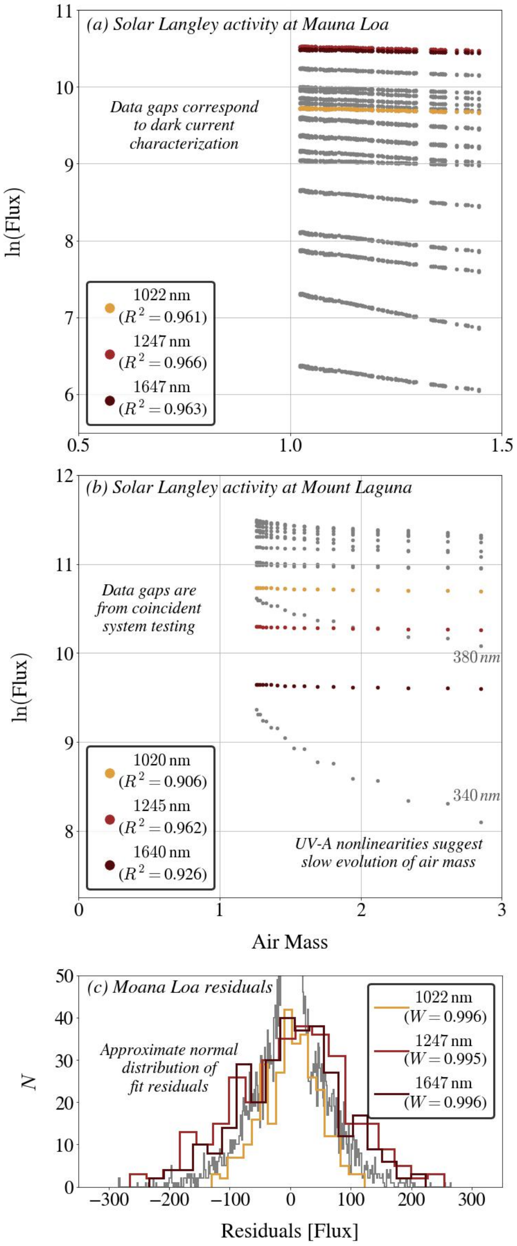

Fig. S1. Sun-pointing radiometric observations obtained during solar Langley calibration activities at Mauna Loa, Hawaii (panel a), and Mount Laguna, California (panel b), respectively. The linear-scale distribution of the residuals to the Langley calibration fit are shown in panel c for the Moana Loa observations. Observations in the UV-A to IR-A (up to 1000 nm) domains are shown in gray, with the two longer IR-A plus the IR-B observations shown in orange, red, and maroon, respectively. Slight changes in the nominal center wavelength between activities reflect instrument updates (Table S1). Panels a and b significantly expand upon limited initial analyses from the third volume (1) of Hooker et al. (2018).

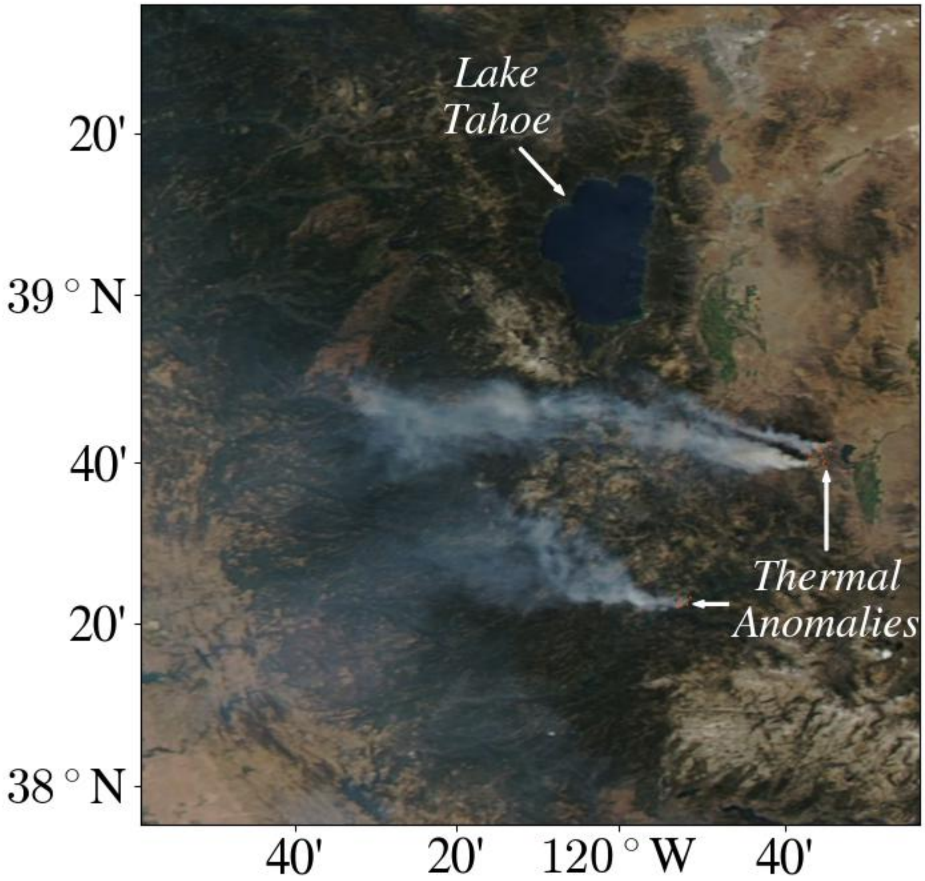

Fig. S2. True-color MODIS Aqua imagery of central eastern California and western Nevada obtained on 1 September 2017 in the vicinity of Lake Tahoe. Active fire regions are determined using MODIS Terra thermal anomalies (`https://worldview.earthdata.nasa.gov/`) and are indicated in orange.

Table S1. **Nominal instrument fixed waveband center wavelengths in nanometers and rounded to the nearest integer value. Spectral configurations were determined based on characteristics of past and future *in situ*, airborne, and satellite missions, atmospheric transmission requirements, and commercial viability in keeping with a COTS instrumentation perspective.**

Photometry		*Above Water*		*In Water*	*Airborne*	Spectral
OSPREy	C-OSPREy	BioSORS	C-AERO	C-HyR	C-AERO	Domain
				313		UV-B
	320		320	320	320	UV-A
341		340	340	340	340	UV-A
380	380	380	380	380	380	UV-A
398		395		395		UV-A
414		412	412	412	412	VIS
444	443	443	443	443	443	VIS
		465		465		VIS
492	490	490	490	490	490	VIS
514		510	510	510	510	VIS
532		532	532	532	532	VIS
558	555	555	555	555	555	VIS
591	589		589	589	589	VIS
627	625	625	625	625	625	VIS
667	670	670	670	670	670	VIS
685		683	683	683	683	VIS
711	710	710	710	710	710	IR-A†
782	780	780	780	780	780	IR-A
	820					IR-A
876		875	875	875	875	IR-A
1022	1020	1020	1020		1020	IR-A
1247	1245	1245	1245		1245	IR-A
1647	1640	1640	1640		1640	IR-B

† Assigned to the IR-A domain for consistency with aquatic optics literature.

References

1. SB Hooker, et al., Advances in above-and in-water radiometry, volume 3: hybridspectral next-generation optical instruments, (National Aeronautics and Space Administration), Technical report (2018).
